# OmpA Binding Mediates the Effect of Antimicrobial Peptide LL-37 on *Acinetobacter baumannii*

**DOI:** 10.1371/journal.pone.0141107

**Published:** 2015-10-20

**Authors:** Ming-Feng Lin, Pei-Wen Tsai, Jeng-Yi Chen, Yun-You Lin, Chung-Yu Lan

**Affiliations:** 1 Department of Medicine, National Taiwan University Hospital Chu-Tung Branch, Hsin-Chu County, Taiwan; 2 Institute of Molecular and Cellular Biology, National Tsing Hua University, Hsin-Chu City, Taiwan; 3 Department of Life Science, National Tsing Hua University, Hsin-Chu City, Taiwan; Nanyang Technological University, SINGAPORE

## Abstract

Multidrug-resistant *Acinetobacter baumannii* has recently emerged as an important pathogen in nosocomial infection; thus, effective antimicrobial regimens are urgently needed. Human antimicrobial peptides (AMPs) exhibit multiple functions and antimicrobial activities against bacteria and fungi and are proposed to be potential adjuvant therapeutic agents. This study examined the effect of the human cathelicidin-derived AMP LL-37 on *A*. *baumannii* and revealed the underlying mode of action. We found that LL-37 killed *A*. *baumannii* efficiently and reduced cell motility and adhesion. The bacteria-killing effect of LL-37 on *A*. *baumannii* was more efficient compared to other AMPs, including human ß–defensin 3 (hBD3) and histatin 5 (Hst5). Both flow cytometric analysis and immunofluorescence staining showed that LL-37 bound to *A*. *baumannii* cells. Moreover, far-western analysis demonstrated that LL-37 could bind to the *A*. *baumannii* OmpA (AbOmpA) protein. An ELISA assay indicated that biotin-labelled LL-37 (BA-LL37) bound to the AbOmpA_74-84_ peptide in a dose-dependent manner. Using BA-LL37 as a probe, the ~38 kDa OmpA signal was detected in the wild type but the *ompA* deletion strain did not show the protein, thereby validating the interaction. Finally, we found that the *ompA* deletion mutant was more sensitive to LL-37 and decreased cell adhesion by 32% compared to the wild type. However, *ompA* deletion mutant showed a greatly reduced adhesion defect after LL-37 treatment compared to the wild strain. Taken together, this study provides evidence that LL-37 affects *A*. *baumannii* through OmpA binding.

## Introduction

Antimicrobial peptides (AMPs) are generated by a wide variety of organisms as a part of the host defense. In humans, AMPs can be produced by various cells and tissues and play a critical role in innate immunity [1,2]. AMPs are generally short (10–100 amino acids), positively charged (normally +2 to +9) and amphiphilic [3]. AMPs can be divided into three major classes based on their gross amino acid composition and certain structural features, including linear alpha-helical peptides (without cysteines), cysteine-containing peptides linked by disulfide bonds and peptides with a high ratio of specific amino acids [2]. For example, human defensins belong to the second class, and histatins are members of the third class. hCAP-18 (the only member of the cathelicidin AMP family in humans) contains an N-terminal domain, a cathelin domain and a C-terminal LL-37 domain [4]. LL-37 is extracellularly cleaved from hCAP-18 by proteinase 3 and belongs to the class of linear alpha peptides. LL-37 owes its name to the fact that it consists of 37 amino acids that begin with two leucine residues [5].

Different types of AMPs use different mechanisms to disrupt bacterial structures or inhibit cell growth [6,7]. For example, the amphipathic conformation change can help an AMP gain access or insert into the plasma membrane of bacteria to disrupt the cells [7]. However, AMPs not only attack membranes but also inhibit cell wall biosynthesis, protein folding, enzyme activity and even protein synthesis through DNA binding [6]. In addition to the direct killing of bacteria, AMPs also play an important role in immunomodulation [8]. AMPs activate the adaptive immune system by stimulating gene transcription to activate macrophages, inducing interleukin-8 in airway epithelial cells to recruit neutrophils, promoting histamine release to increase blood vessel permeability, activating fibroblast growth to facilitate wound healing and presenting chemotactic activity to recruit monocytes [1,9]. These multi-functional responses induced by AMP make it a promising candidate adjuvant therapeutic agent, especially against multidrug-resistant pathogens.

Human LL-37 is able to defend against various bacterial and fungal pathogens [10–12]. Recently, *Acinetobacter baumannii* has emerged as an important pathogen in nosocomial infections [13]. Infections and outbreaks caused by multidrug-resistant *A*. *baumannii* (MDRAB) are rapidly increasing [14]. Resistance to the last resort antibiotics for carbapenem-resistant *A*. *baumannii*, including tigecycline and colistin, has been reported [15,16]. A previous study reported a lipopolysaccharide (LPS)-deficient, colistin-resistant *A*. *baumannii* strain that showed reduced viability even at a low concentration of LL-37 [17]. Moreover, LL-37 and its fragments possess both antimicrobial and antibiofilm activities against MDRAB [18]. Therefore, human antimicrobial peptides (especially LL-37) may function as potential therapeutic alternatives or adjuvants to antibiotics.

The OmpA outer membrane protein of *Escherichia coli* and other enterobacteria is a multifaceted protein, which functions as an adhesin and invasin, participates in biofilm formation, acts as both an immune target and evasin, and serves as bacteriophage receptor [19]. The *A*. *baumannii* outer membrane protein A (AbOmpA) is a trimeric porin that is involved in solute transport and virulence [20]. The contributions of AbOmpA to pathogenesis include apoptosis, immunomodulation, cell adherence and invasion, biofilm formation and serum resistance. AbOmpA can induce dendritic cell death via targeting to the mitochondria [21]. Interaction of laryngeal epithelial cells with AbOmpA has a significant impact on the induction of innate immunity during the early stages of *A*. *baumannii* infection [22]. AbOmpA also plays a role in biofilm formation on abiotic surfaces [23]. Serum resistance to *A*. *baumannii* occurs through binding of factor H to outer membrane proteins (OMPs), including OmpA [24]. Because AbOmpA is multi-functional, we hypothesize that it may also bind to LL-37. Therefore, the aim of this study is to determine the effect of LL-37 on *A*. *baumannii* and to determine whether the effect was mediated via binding to OmpA.

## Materials and Methods

### Peptides, *A*. *baumannii* Strains, Media, and Growth Conditions

LL-37 (LLGDFFRKSKEKIGKEFKRIVQRIKDFLRNLVPRTES), biotin-labeled LL-37 (BA-LL37), biotin-labeled human β-defensin-3 (BA-hBD3, QKYYCRVRGGRCAVLSCLPKEEQIGKCSTRGRKCCRRKK), biotin-labeled histatin 5 (BA-Hst5, DSHAKRHHGYKRKFHEKHHSHRGY), OmpA_164-181_ (TYNADEEFWNYTALAGLN) and OmpA_74-84_ (GDVDGASAGAE) were synthesized by MDBio, Inc. (Taipei, Taiwan). The purity of these peptides was determined to be ≧85% by high performance liquid chromatography (HPLC) and mass spectrometry. The *A*. *baumannii* ATCC 17978 strain was used as the wild type. The media and growth condition were the same as described in our previous study [25].

### Assays for LL-37 Anti-*Acinetobacter* Activity

Bacterial cells were grown overnight (for ~16 h) in LB broth and subcultured into 5 ml of fresh LB medium (initial OD_600_ ~0.27). Then, the cells were grown to an OD_600_ of 1.2 and harvested by centrifugation (6000 x g). Cell pellets were washed twice and re-suspended with phosphate-buffered saline (PBS). Different concentrations of LL-37, BA-LL37, BA-hBD3 or BA-Hst5 were incubated with the cells (1 X 10^7^ cells/ml) in 750 μl of RPMI-1640 medium at 37°C with 5% CO_2_ for 30 min. After the incubation, the cells were serially diluted 10-fold with PBS, spotted onto LB agar plates (10 μl/per spot) and incubated at 37°C overnight. To determine colony forming units (CFUs), the cells were 10-fold serially diluted, and 100 μl of each sample was plated onto LB agar plates.

### *A*. *baumannii* Adhesion Assay

The adhesion of *A*. *baumannii* was assessed as previously described with some modifications [26]. Briefly, bacterial cells were grown overnight in LB broth (~16 hr) and subcultured into 25 ml of fresh LB medium (initial OD_600_ ~0.35). The cells were grown to an OD_600_ of 1.2 and harvested by centrifugation (6000 x g). The cell pellets were washed twice and re-suspended with PBS. Different concentrations of LL-37 were incubated with the cells (4 X 10^8^ cells/ml) in each well of a 96-well plate (Nunc^TM^, Rochester, NY, USA) as previously described [26]. After incubation at 37°C for 1 hr with shaking (100 rpm), the non-adherent floating cells were discarded, and the adherent cells were washed three times with PBS. A total of 150 μl of crystal violet was added to each sample, and the plates were incubated at room temperature for 20 min. After removal of the crystal violent solution, each sample was washed three times with double-distilled water (ddH_2_O). The remaining crystal violet in each well was dissolved in 100 μl of 95% ethanol, and the absorbance at 595 nm was detected using an iMARK microplate reader (Bio-Rad Life Science, Hercules, CA, USA).

### *A*. *baumannii* Motility Assay

The motility assay was performed as previously described [27]. Cells from an overnight culture were subcultured into 5 ml of fresh LB medium and grown to an OD_600_ of 1.2. The cells were harvested by centrifugation (6000 x g), and a 5 μl cell suspension (~1 X 10^9^ cells) was spotted onto motility agar (1% tryptone, 0.5% NaCl and 0.4% agarose) and incubated at 37°C for 10 hr.

### Flow Cytometric Analysis

Cells from an overnight culture were inoculated into 5 ml of fresh LB medium (initial OD_600_ 0.27) and grown to an OD_600_ of 1.2. Cell pellets were harvested by centrifugation (6000 x g), washed twice with PBS and re-suspended with 750 μl of ice-cold PBS (containing 5 X 10^6^ cells). Then, the cells were incubated overnight at 4°C with or without different concentrations of BA-LL37, BA-LL37, BA-hBD3, or BA-Hst5. Binding of the BA-AMPs to the bacterial cells was assessed by flow cytometry based on SA-4,6-dichlorortriazinyl aminofluorescein (SA-DTAF) detection. Three microliters of SA-DTAF was used in each reaction (Jackson ImmunoResearch, West Grove, PA, USA). Reactions were quantified using a FACSCalibur flow cytometer (BD Biosciences, Franklin Lakes, NJ, USA) according to a previously described method [28]. Fluorescence data for 2 X 10^4^ cells were acquired per experiment.

### Immunofluorescence Staining

Bacterial cells from overnight cultures were subcultured into 5 ml of fresh LB medium (initial OD_600_ ~0.27). Cells were grown to an OD_600_ of 1.2 and harvested by centrifugation (6000 x g). The cells were re-suspended with PBS to an OD_600_ of 1, mixed with or without BA-LL37 (20 μg/ml) to a final volume of 750 μl, and grown overnight at 4°C. Next, the cell pellets were harvested by centrifugation (6000 x g), washed twice with ice-cold PBS and re-suspended in 750 μl of ice-cold PBS containing SA-DTAF (3 μg/reaction). The mixture was incubated at 4°C for 2 hr. Finally, the cell pellets were collected by centrifugation and re-suspended in 40 μl of ice-cold PBS. The cell suspension was then transferred to a cover slip at 4°C for 20 min. The samples were examined with a Carl Zeiss AXIO IMAGER A1 Microscope.

### Extraction of *A*. *baumannii* Outer Membrane Proteins (AbOMPs)

Extraction of AbOMPs was performed as previously described [29] with some modifications. Briefly, *A*. *baumannii* cells were grown overnight, subcultured into 100 ml of fresh LB medium and incubated at 37°C with shaking (220 rpm) for 2 hr. The cell pellets were harvested by centrifugation (6000 x g at room temperature) and washed twice with PBS. Then, the cells were re-suspended in 20 ml of RPMI-1640 medium and incubated at 37°C with shaking (100 rpm) for 1 hr. The cell pellets were washed twice with PBS and re-suspended in 20 ml of 10 mM phosphate buffer (pH7.2) supplemented with phenylmethanesulfonylfluoride (PMSF) at a final concentration of 1 mM. The cells were disrupted by sonication for 12 min on ice (10 sec sonication at intervals of 10 sec). The cell debris was discarded by centrifugation (3000 x g), and the supernatant was subjected to centrifugation at 13,700 x g (4°C, 45 min). Then, the supernatant was discarded, and the extracted proteins were solubilized at room temperature using 2% sodium lauryl sarcosinate (Sarkosyl) in 10 mM phosphate buffer for 30 min. Finally, the AbOMPs were collected by centrifugation at 13,700 x g (4°C, 45 min), re-suspended in 62.6 mM Tris-HCl buffer, and stored at -20°C.

### Western and Far-Western Analysis

AbOMP extracts were mixed with sample buffer and heated at 100°C for 10 min. OMP samples were separated by 12% sodium dodecyl sulfate-polyacrylamide gel electrophoresis (SDS-PAGE) and transferred onto a polyvinylidene difluoride (PVDF) membrane (Pall Corporation, Port Washington, NY, USA) using a TE77 ECL Semi-Dry Transfer Unit (Amersham Biosciences). The membrane was blocked with 3% non-fat milk in PBST (PBS with 1% Tween 20) at room temperature for 1 hr and washed with PBST. Then, the membrane was hybridized overnight at 4°C with anti-OmpA (1:5000; a kind gift from Luis A. Actis, Miami University, OH, USA) in PBST containing 1% BSA. The blotted membrane was washed with PBST for 25 min and probed with anti-rabbit IgG (1:5000; GeneTex) at room temperature for 40 min. The membrane was again washed with PBST for 25 min. The Western Lightning Plus-ECL reagent (PerkinElmer Life Science, SC-2004) and an ImageQuantTM LAS 4000 mini system (GE Healthcare Science) were used to detect proteins immobilized on the membrane according to the manufacturer’s instructions.

For the far-western analysis, outer membrane extraction and separation were performed as described above for the western analysis. The membrane was blocked with 3% bovine serum albumin in PBST at room temperature for 2 hr and washed with PBST. Then, the membrane was hybridized overnight with 10 μg/ml of BA-LL37 (in PBST) at 4°C. Proteins were detected using the Western Lightning Plus-ECL reagent and an ImageQuantTM LAS 4000 mini system.

### Coomassie Blue Staining

After SDS-PAGE, the gel was soaked overnight in the fixing buffer (35% ethanol and 2% phosphoric acid) at room temperature. Next, the gel was washed with water for 90 min at room temperature, with water changes every 30 min. After washing, the gel was soaked in 50 ml of staining buffer (34% methanol, 17% (NH_4_)_2_SO_4_, and 3% phosphoric acid) for 1 hr at room temperature. Finally, 25 mg of Coomassie Blue G-250 was dissolved into 50 ml of the staining buffer. The gel was stained in the solution until blue colored bands appeared.

### Prediction of LL-37 Binding Sites within the AbOmpA Protein and ELISA Assay to Investigate LL-37 Binding to the AbOmpA_74-84_ and AbOpmA_164-181_ Peptides

Based on our previous report [26], ΦHWXΦXΦXΦ (Φ: a hydrophobic amino acid residue; X: any amino acid residue) is a consensus sequence derived from different peptides that can bind LL-37. This sequence was used to blast search for possible LL-37 binding sites within the AbOmpA protein. Members of the OmpA family of bacteria are known to commonly contain four surface-exposed loop structures [30]. Amino acid sequences representing these structures were identified within the AbOmpA protein. We found that the amino acid residues between 74 to 84 and 164 to 181 of the AbOmpA protein matched the consensus sequences for LL-37 binding and were possibly located on two independent loops exposed towards the outside of the outer membrane. Therefore, the two peptides AbOmpA_74-84_ and AbOmpA_164-181_ were used to facilitate the following experiments.

Both AbOmpA_164-181_ (TYNADEEFWNYTALAGLN) and AbOmpA_74-84_ (GDVDGASAGAE) were synthesized by MDBio, Inc. (Taipei, Taiwan). The peptides were mixed with carbonate-bicarbonate coating buffer (30 mM Na_2_CO_3_ and 69 mM NaHCO_3_, pH 9.6) as previously described [31]. A total of 5~10 μg of AbOmpA_74-81_ or AbOmpA_164-181_ in 100 μl of carbonate-bicarbonate coating buffer was transferred into a Nunc Maxisorp 96-well plate (Nunc^TM^, Rochester, NY, USA) and immobilized at 4°C overnight. After peptide immobilization, each well was washed three times with PBST to remove non-attached peptides. For blocking, 100 μl PBST and 2% BSA were added to each well; the mixture was incubated at room temperature for 1 hr, followed by removal of the blocking reagents. Each well was washed three times with PBST. Different concentrations of BA-LL37 (in 100 μl of PBS) were added to each well and incubated at room temperature for 1 hr. Unbound BA-LL37 was removed, and the wells were washed three times with PBST. Finally, SA-HRP (1:200 in PBST) was added to each well and incubated at room temperature for 30 min. After removing the SA-HRP solution, each well was washed three times with PBST. For signal detection, 100 μl of 3,3',5,5'-tetramethylbenzidine (TMB) was added to each well until the blue color began to appear. Then, 100 μl of 1N H_2_SO_4_ was added to stop the reaction, and the signal was detected at OD_450_ using an iMARK ELISA reader (BioRad).

### Genomic DNA Extraction, RNA Isolation and Reverse Transcription (RT)-PCR

Genomic DNA extraction, RNA isolation and RT-PCR were performed as described in our previous work [25]. For genomic DNA extraction, cells from an overnight culture (1 ml) were harvested by centrifugation (6000 x g) for 1 min and then mixed with 600 μl of lysis solution (200 mM Tris-HCl [pH 8.5], 100 mM EDTA [pH 8.0], and 35 mM SDS). Cells were lysed in an 80°C water bath for 10 min; then, 200 μl of 10 M NH_4_OA_C_ was added, and the samples were vortexed vigorously for 20 sec. The mixture was centrifuged (16500 x g, 4°C) for 5 min, and the supernatant (650 μl) was mixed with an equal volume of ice-cold PCIA (phenol [pH 7.0]-chloroform-isoamyl alcohol [25:24:1, v/v]). After centrifugation (16500 x g, 4°C) for 5 min, 500 μl of the supernatant was mixed with an equal volume of isopropanol to precipitate the DNA. DNA pellets were collected by centrifugation (16,500 x g, 4°C) for 5 min, washed with 75% ethanol and centrifuged again. The pellets were re-suspended with 400 μl of water containing 75 μg/ml RNase A and incubated at 37°C for 15 min. Then, the sample was mixed with two volumes of 99.5% ethanol and a 1/10 volume of 3 M ammonium acetate to precipitate the DNA. The sample was centrifuged at 16,500 x g at 4°C for 5 min, and the DNA pellets were re-suspended with 100 μl of water.

To extract total RNA, cells were grown in LB broth to the mid-log phase and harvested by centrifugation at 4°C. Cell pellets were re-suspended in 200 μl of ice-cold RNA extraction buffer (0.1 M Tris-HCl [pH 7.5], 0.1 M LiCl, 0.01 M EDTA [pH 8.0], 5% SDS, and 2% β-mercaptoethanol) and 200 μl of ice-cold PCIA (pH 4.5). The extraction was repeated three times, and the extracts were collected by centrifugation. Two volumes of ethanol (pre-cooled at -20°C) and 0.1 volumes of 3 M NaOAc were added to precipitate the RNA overnight at -80°C. RNA was pelleted by centrifugation at maximum speed (5 min) and re-suspended in 25–100 μl of DEPC-treated water. DNA contaminants were removed using Ambion® TURBO™ DNase. cDNAs were synthesized using the High-Capacity cDNA Reverse Transcriptase Kit (Applied Biosystems).

### Construction of an *ompA* Deletion (Δ*ompA*) Mutant of *A*. *baumannii*

To determine the involvement of OmpA in LL-37 binding, the *ompA* gene (A1S_2840) was deleted and replaced with a kanamycin resistance (*Kan* ^*R*^) gene using the pEX18Tc plasmid [25]. Briefly, 1000 bp flanking sequences upstream or downstream of the *ompA* gene were independently PCR-amplified from the genomic DNA of the *A*. *baumannii* ATCC 17978 strain. The primer pairs used for amplification of the *ompA*-upstream sequences were ompA5’F (the *Sal*I site is underlined) and ompA5’R (the *Bam*HI site is double underlined; see S1 Table). The primer pairs used for amplification of the *ompA*-downstream sequences were ompA3’F (the *Kpn*I site is underlined) and ompA3’R (the *Sac*I site is double underlined). The PCR products containing the *ompA* upstream and downstream flanking regions were digested with *Sal*I/*Bam*HI and *Kpn*I/*Sac*I and independently cloned into pEX18Tc, generating pEX18Tc-ompAUD. The *Kan* ^*R*^ gene was obtained by PCR amplification using the TOPO^®^ vector as a template [32] and the primer pairs *kanF* (the *BamH*I site is underlined) and *kanR* (the *Kpn*I site is double underlined). The PCR product carrying *Kan* ^*R*^ was digested with *Bam*HI and *Kpn*I and cloned into the *Bam*HI/*Kpn*I sites of pEX18Tc-ompAUD to generate pEX18Tc-OmpAUD-Kan. To construct the *ompA* deletion mutant, pEX18Tc-ompAUD-Kan was transformed into the *E*. *coli* S17-1 λ-pir strain. The *E*. *coli* transformant and *A*. *baumannii* ATCC 17978 strain were grown separately overnight and mixed (1:3) in 1 ml of fresh LB medium. The cell mixture was further incubated at 37°C for 1 hr. Then, 20 μl of the cell mixture was spotted onto an LB agar plate and incubated overnight at 37°C to obtain cells with a single gene crossover. To eliminate *E*. *coli* from the mixed culture, the mixture was grown overnight in 5 ml of LB medium containing 100 μg/ml ampicillin. To obtain cells with two gene crossovers, the mixed culture was pelleted and plated onto LB agar plate containing 10% sucrose and kanamycin (50 μg/ml) at 37°C for 16 hr. The sucrose-resistant colonies were picked, and the *ompA* deletion was verified by PCR and validated by RT-PCR and SDS-PAGE.

## Results

### LL-37 Kills *A*. *baumannii* in a Dose-Dependent Manner

**Fig 1:**
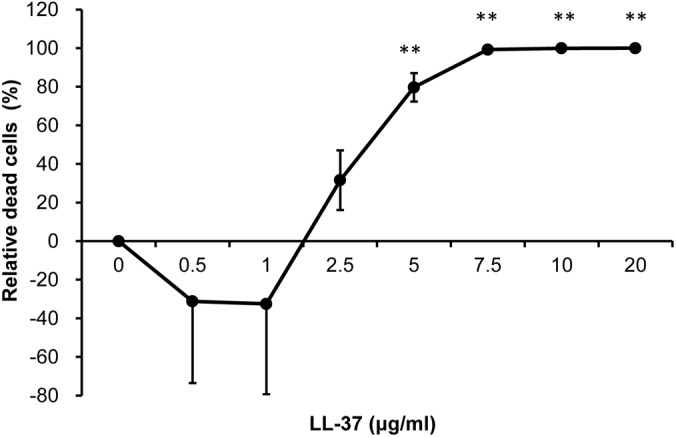
Anti-*Acinetobacter* activity of LL-37. Anti-*Acinetobacter* activity of LL-37 was determined by counting CFUs. The CFU counts of cells (1 X 10^7^ cells/ml) in 750 μl of RPMI-1640 medium treated with different concentrations of LL-37 were normalized to the control without LL-37 treatment and presented as a percentage. The result indicated that LL-37 exhibited a bactericidal effect on *A*. *baumannii*. These experiments were performed in triplicates (N = 3). The Student’s t-test was used to determine (**, p<0.01) the statistical significance of the experimental data.

To assess the anti-*Acinetobacter* activity of LL-37, bacterial cells were treated with different concentrations of LL-37; viable cells were counted and represented as the number of CFUs. In Fig 1, the results indicated that LL-37 harbored dose-dependent bactericidal activity against *A*. *baumannii*. Approximately 32%, 80%, and 99% of the cells died following treatment with 2.5, 5 and 7.5 μg/ml of LL-37, respectively. Moreover, no viable cells were apparent after treatment with 20 μg/ml of LL-37 (Fig 1). The bacterial killing activity of LL-37 on two clinical isolates of *A*. *baumannii* was also performed using spot assay, which showed the anti-bacterial effect augmented with LL-37 concentrations increasing (S1 Fig). These results indicate that LL-37 exhibits bactericidal activity against *A*. *baumannii*.

### LL-37 Inhibits *A*. *baumannii* Motility and Adhesion

**Fig 2:**
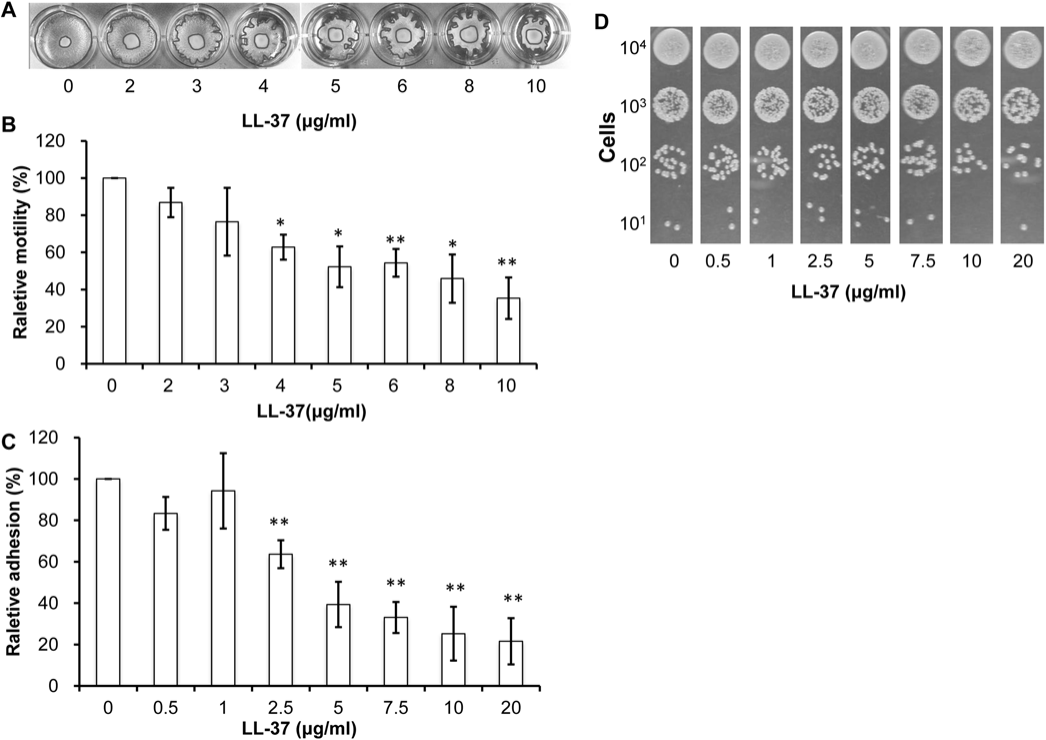
Inhibition of *A*. *baumannii* adhesion and motility by LL-37. (A) Motility of *A*. *baumannii* on semisolid agar. Three microliters of cells from mid-log phase were spotted onto the middle of semisolid agar containing different concentrations of LL-37 and incubated at 37°C for 7 hr. (B) Cell motility was quantified using the Image J program (http://imagej.nih.gov/ij/). The whole area of the entire colony and the original spot was measured. Cell motility was obtained by subtracting the area of the original spot from the area of the entire colony. The relative percentage of motility was obtained by comparing the motility of cells treated with LL-37 to of the motility of the control without LL-37 treatment. The results of both (A) and (B) showed that *A*. *baunmannii* motility was reduced by increasing concentrations of LL-37. (C) Cell adhesion to a polystyrene surface. Cells were mixed with different concentrations of LL-37 in RPMI-1640 and incubated at 37°C for 1 hr. Adherent cells were detected by crystal violet staining, and relative adhesion was represented as a percentage. (D) Non-adherent floating cells were collected by centrifugation, serially diluted and spotted onto an LB agar plate. The results indicated that *A*. *baumannii* cell attachment was decreased with treatment of increasing concentrations of LL-37 and that most non-adherent cells were alive. Both adhesion and motility experiments were performed in triplicates (N = 3). The Student’s t-test (**, p<0.01) was used to determine the statistical significance of the experimental data.

Although *A*. *baumannii* is generally considered to be “non-motile”, several studies indicated that the *A*. *baumannii* ATCC 17978 strain had the ability to migrate under certain conditions [27,33]. Moreover, motility was recently identified as an *A*. *baumannii* virulence factor [27]. To test the effect of different concentrations of LL-37 on *A*. *baumannii* motility, cells were grown to the exponential phase and spotted onto motility agar plates (Fig 2A). A reduction in *A*. *baumannii* motility was observed concomitant with treatment with increasing concentrations of LL-37 (Fig 2B).

Adhesion is another *A*. *baumannii* virulence factor [34]. To determine the effects of LL-37 on *A*. *baumannii* adhesion, cells were grown in a 96-well microplate and adherent cells were quantified by crystal violet staining. We found that *A*. *baumannii* cell attachment was decreased with increasing concentrations of LL-37 (Fig 2C). To determine whether the inhibition of bacterial adhesion by LL-37 was due to bacterial cell death, the floating non-adherent cells were serially diluted and spotted onto LB agar plates. As shown in Fig 2D, bacterial growth was not significantly affected among the floating cells treated with different concentrations of LL-37. Therefore, LL-37-mediated inhibition of adhesion was not a consequence of LL-37-induced cell death.

### LL-37 Binds to *A*. *baumannii* Cells

**Fig 3:**
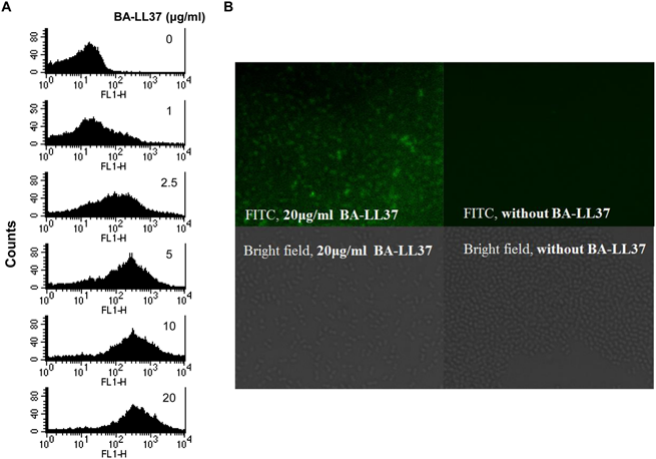
BA-LL37 binding to *A*. *baumannii*. (A) LL-37 binding to *A*. *baumannii* cells was determined by flow cytometry. Cells were incubated with different concentrations of BA-LL37 at 4°C in ice-cold PBS overnight. The binding between BA-LL37 and *A*. *baumannii* was detected by adding SA-DTAF. The increase of signal was detected and correlated to cells treated with increasing concentrations of LL-37, indicating that LL-37 binds directly to *A*. *baumannii*. These data are representative of three independent experiments with similar results. FL1-H indicates the extent of the fluorescence intensity. (B) An immunofluorescence staining assay was performed to verify LL-37 binding to *A*. *baumannii*. In the presence of 20 μg/ml LL-37, fluorescence was observed compared to the control (without BA-LL37 treatment). Samples were observed using a Carl Zeiss AXIO IMAGER A1 microscope.

In many cases, the first step in the AMP bacterial killing ability is to bind to the bacterial cell surface [6]. Therefore, we hypothesized that LL-37 might directly bind to the cell surface. To test this hypothesis, we used a flow cytometric assay based on SA-DTAF detection (as described in the Materials and Methods). An increase in the fluorescence intensity was correlated with increasing concentrations of BA-LL-37, indicating that LL-37 could directly bind to *A*. *baumannii* cells (Fig 3A). Immunofluorescence staining was also performed. *A*. *baumannii* cells were incubated with BA-LL37 overnight and stained with SA-DTAF. In the presence of 20 μg/ml LL-37, increased fluorescence was observed compared to the control cells without BA-LL37 treatment (Fig 3B). Therefore, these studies showed that LL-37 bound to *A*. *baumannii* cells.

### LL-37 Is Highly Efficient against *A*. *baumannii* Compared to Other AMPs

**Fig 4:**
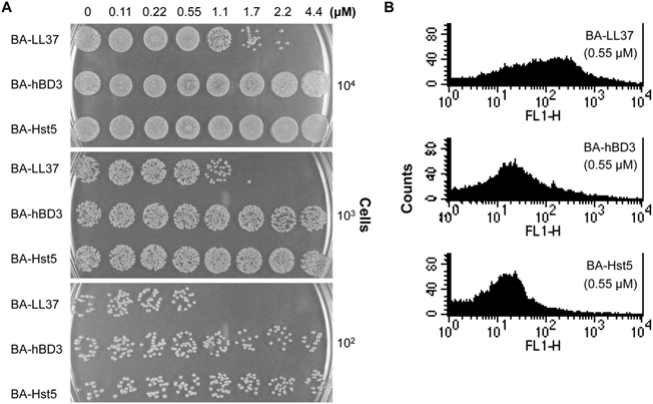
Efficient action of LL-37 on *A*. *baumannii*. (A) A spot assay was performed to compare the anti-*Acinetobacter* activities of three different AMPs. The cells were treated with different concentrations of BA-LL37, BA-hBD3 and BA-Hst5 for 30 min, ten-fold serially diluted and then spotted onto LB agar plates. BA-LL37 showed a more efficient killing of *A*. *baumannii* than BA-hBD3 or BA-Hst5. The data are representative of three independent experiments with similar results. (B) Binding of different AMPs to *A*. *baumannii* was examined using flow cytometry. Cells were incubated with 0.55 μM of each BA-AMP (in ice-cold PBS) at 4°C overnight, and the attachment of BA-AMP to the cells was analyzed by flow cytometry based on SA-DTAF detection. FL1-H represents the extent of the fluorescence intensity. Compared to BA-LL37, BA-hBD3 and BA-Hst5 showed poor binding to *A*. *baumannii*. The image is representative of three independent experiments with similar results.

There are various AMPs in the human immune system, including LL-37, hBD3 and Hst5 [2]. To compare their anti-*Acinetobacter* activity, cells were treated with different concentrations of these three AMPs and spotted onto LB agar plates. Fig 4A showed that LL-37 killed *A*. *baumannii* cells more efficiently than hBD3 or Hst5. Additionally, the ability of hBD3 and Hst5 to bind to *A*. *baumannii* was determined by flow cytometric analysis. Compared to LL-37, hBD3 and Hst5 exhibited much poorer binding to *A*. *baumannii* when the same concentration of each AMP was used (Fig 4B). We concluded that the effects of LL-37 on *A*. *baumannii* were more efficient compared to hBD3 and Hst5.

### AbOmpA Is a Binding Target of LL-37

**Fig 5:**
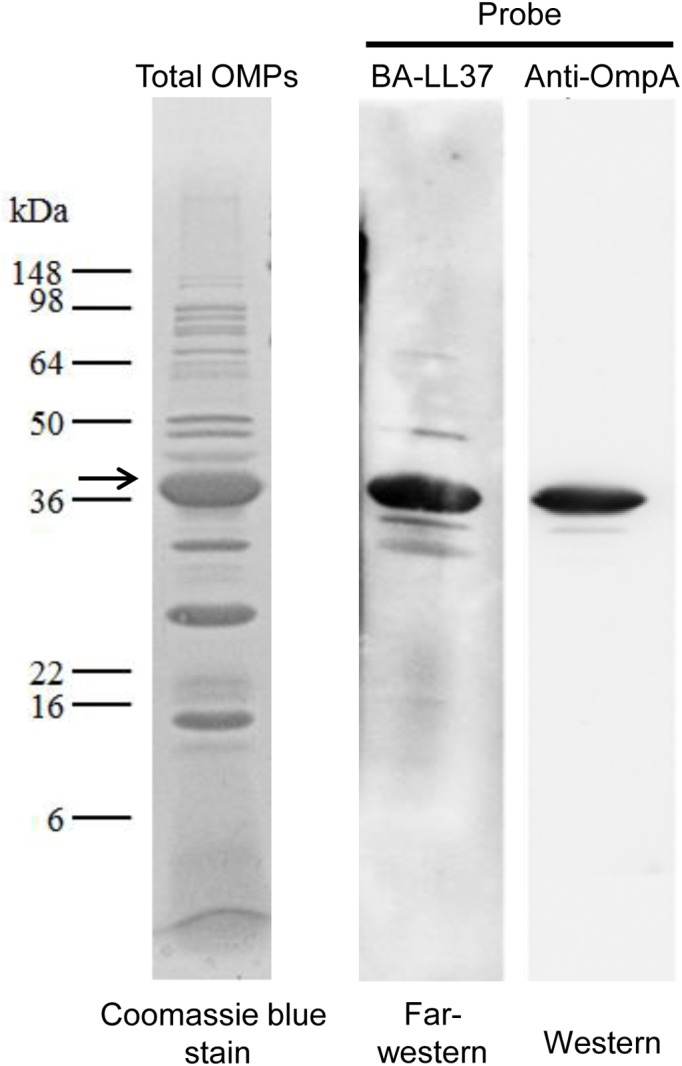
LL-37 binding to *A*. *baumannii* OmpA. Total OMPs of *A*. *baumannii* were isolated, subjected to 12% SDS-PAGE and detected by western blotting. After electrophoresis, OMPs were stained with Coomassie blue (Lane 1). OMPs in another gel were transferred onto a PVDF membrane using a semi-dry transfer system and blotted with SA-HRP (Lane 2) or an anti-OmpA antibody (Lane 3). The signals were detected using enhanced chemiluminescence (ECL).

Because LL-37 bound to *A*. *baumannii* cells, we were interested in identifying potential target(s) for LL-37 on the cell surface, and particularly on the *A*. *baumannii* outer membrane. OMPs were isolated from *A*. *baumannii* [29], immediately subjected to SDS-PAGE, and transferred onto a PVDF membrane (Fig 5, Lane 1). Far western analysis was performed using BA-LL37 as a probe. Several OMPs bound BA-LL37, with a protein with a molecular mass of ~38 kDa showing the strongest signal (Fig 5, Lane 2). The molecular mass of 38 kDa is close to that of AbOmpA, an *A*. *baumannii* outer membrane porin protein. To determine whether the protein was indeed AbOmpA, western blotting was performed using an anti-AbOmpA antibody. As shown in lane 3 of Fig 5, a protein of ~38 kDa was detected. These results suggest that AbOmpA is an LL-37 binding target.

### LL-37 Associates with Certain Regions of AbOmpA

**Fig 6:**
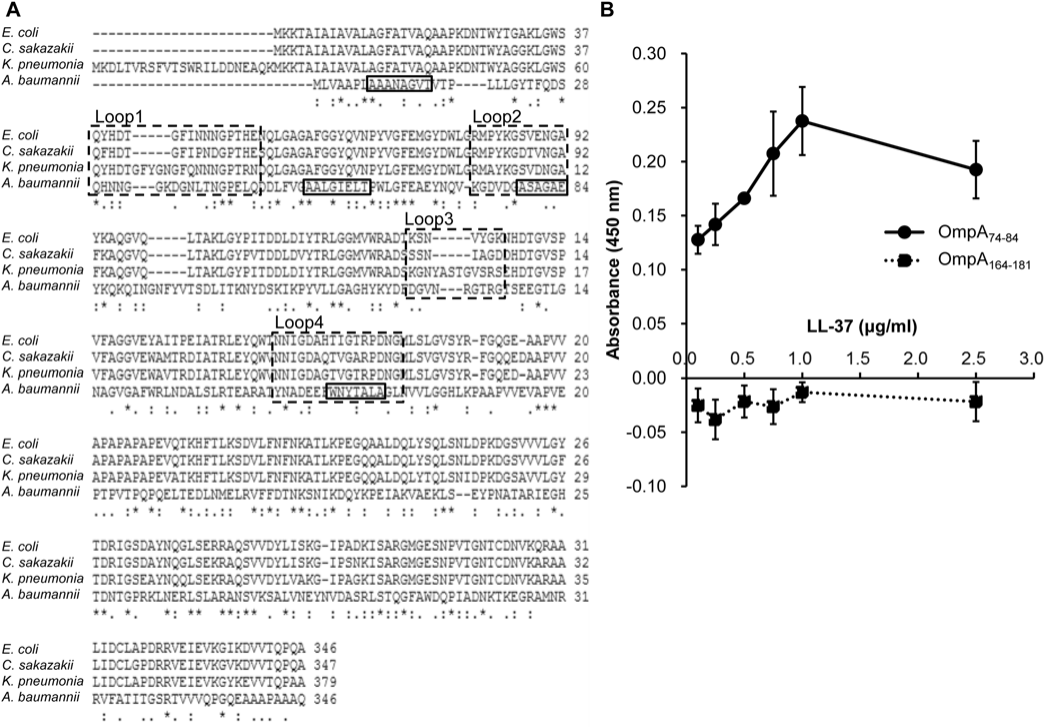
Detection of binding between OmpA peptides and LL-37. (A) Amino acid sequence alignment of OmpA from *E*. *coli*, *Klebsiella pneumoniae*, *Cronobacter sakazakii* and *A*. *baumannii*. The alignment was performed using Clustal W2. The conserved Loop 1 to Loop 4 were boxed in dashed lines, and four potential LL-37-binding sequences were boxed in solid lines. (B) ELISA assay for binding of the AbOmpA_74-84_ and AbOmpA_164-181_ peptides to LL-37. Each peptide was mixed with carbonate coating buffer and immobilized on each well of a microplate at 4°C overnight and then washed with PBS. After blocking, various concentrations of BA-LL37 were added to the immobilized peptides and the binding activity was detected with SA-HRP. The results indicated that BA-LL37 could bind to the AbOmpA_74-84_ peptide, but not the AbOmpA_164-181_ peptide, in a dose-dependent manner. All assays were performed in triplicate and repeated three times.

Using phage display screening of a linear dodeca-peptide library, several peptide sequences associated with LL-37 were identified in a previous study from our laboratory [26]. From these identified LL-37-binding peptides, a conserved sequence of ΦHWXΦXΦXΦ (Φ: a hydrophobic residue; X: any residues) was proposed. Additionally, sequence analysis of AbOmpA revealed 4 loops that were highly conserved in the OmpA protein family of Gram-negative bacteria (Loops 1 to 4, boxed in dashed lines, Fig 6A) [30]. To determine whether AbOmpA was truly an LL-37-binding target, the consensus peptide sequence was used to blast search against the entire protein sequence of AbOmpA. The conserved sequence derived from the phage display assay aligned with four regions of the AbOmpA protein (boxed in solid lines, Fig 6A). Two of the regions (AbOmpA amino acid residues 74–84 and 164–181) were located within Loops 2 and 4, respectively (Fig 6A). Therefore, two peptides (AbOmpA_74-84_ and AbOmpA_164-181_) were synthesized and used in an ELISA assay to examine their association with LL-37. The results indicated that BA-LL37 could bind to the AbOmpA_74-84_ peptide in a dose-dependent manner (Fig 6B). However, the AbOmpA_164-181_ peptide did not bind to BA-LL37. These results raise the possibility that LL-37 binding to AbOmpA may be dependent on the recognition of specific region(s).

### LL-37-AbOmpA Interaction

**Fig 7:**
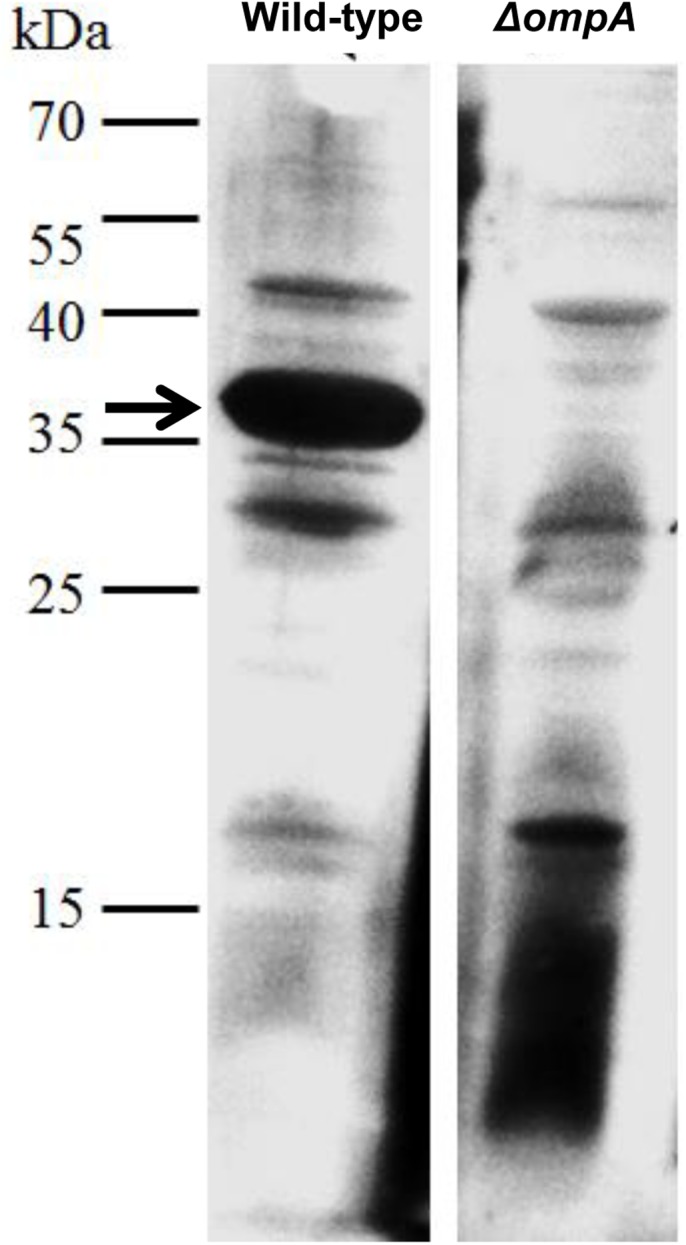
LL-37 and AbOmpA interaction. Binding of LL-37 to the wild type and ΔompA strains was determined by far-western analysis. OMPs were isolated and subjected to SDS-PAGE. The proteins were transferred onto a PVDF membrane, followed by probing with an anti-OmpA antibody. The ~38 kDa OmpA was detected in the wild type strain but was absent in the Δ*ompA* mutant.

To verify the finding that LL-37 bound to AbOmpA, we constructed an *ompA* deletion mutant by gene displacement. The successful construction of the mutant was confirmed by RT-PCR and western blot analysis (S2 Fig). Then, a far western assay was performed to compare BA-LL37 binding to the wild type and *ompA* deletion strain. Using BA-LL37 as a probe, the ~38 kDa OmpA was detected in the wild type but absent in the Δ*ompA* strain (Fig 7), thereby confirming that LL-37 indeed bound to AbOmpA.

### AbOmpA Influences LL-37’s Effect on Bacterial Killing and Bacterial Adhesion

**Fig 8:**
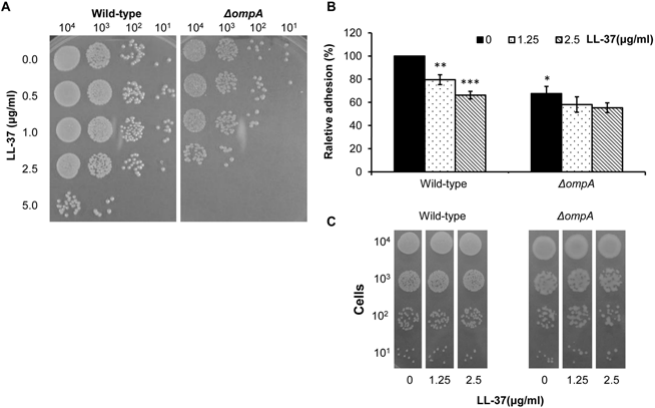
Comparison of LL-37 sensitivity and adhesion ability between the wild type and Δ*ompA* strains. (A) Sensitivity of the wild-type and Δ*ompA* strains to LL-37 killing was examined using the spot assay. Cells were incubated with different concentrations of LL-37 for 1 hr. Then, the cells were 10-fold serially diluted and spotted onto LB agar plates. The result showed that the Δ*ompA* mutant was more sensitive to LL-37 than the wild type. (B) Cell adhesion of the wild type and Δ*ompA* strains was compared. The wild type and Δ*ompA* cells were attached to polystyrene for 1 hr. Then, non-adherent cells were removed by centrifugation and the adherent cells were stained with crystal violet. The Δ*ompA* mutant showed a decrease in adhesion of 32% compared to the wild type. The adhesion defect in the Δ*ompA* strain was not augmented as obviously as that of the wild type after the addition of LL-37. (C) The spot assay demonstrated that the difference in bacterial adhesion induced by LL-37 was not due to bacterial cell death. The Student’s t-test (**p <0.01) was used to determine the statistical significance of the experimental data.

To determine the influence of AbOmpA on LL-37-mediated bacteria-killing, the sensitivity of the Δ*ompA* strain to LL-37 was examined using a spot assay. The result showed that the Δ*ompA* strain was more sensitive to LL-37 than the wild type strain (Fig 8A). This result was unexpected. Because bacterial OmpA interacts with lipopolysaccharide (LPS) and both OmpA and LPS are major components of the outer membrane [35,36], we used the spot assay to compare LL-37 susceptibility between the wild type strain and an LPS-defective mutant (S3 Fig). The result indicated that the LPS-defective mutant had a better tolerance to LL-37 than the wild type. Moreover, the adhesion ability of the wild type and Δ*ompA* strains was compared with or without LL-37 treatment. The Δ*ompA* strain showed a decrease in adhesion of 32% compared to the wild type strain without LL-37 treatment (Fig 8B). After *ompA* deletion, the effect of LL-37 on adhesion was greatly reduced compared to the wild strain. Bacterial growth was not significantly different between the floating cells treated with 1.25 and 2.5 μg/ml of LL-37 (Fig 8C). This result implies that LL-37 may impair the adhesion of *A*. *baumannii* through binding to OmpA.

## Discussion

LL-37 is an important component of the human innate immune defense [5,37]. LL-37 not only plays a critical role in bacterial clearance but also regulates host activities related to the immune response, including chemotactic migration and wound healing. The cationic, α-helical peptide LL-37 efficiently kills both Gram-positive and Gram-negative bacteria. Besides, the discovery of LL-37-inducing components, such as butyrate and vitamin D(3), has opened new avenues to prevent or treat infections by boosting innate immune response [38,39]. Although different approaches (e.g., bulk assay, model membrane assay and minimum inhibitory concentration test) have been used and different mechanisms have been proposed for the activity of LL-37 against bacteria [5,40], the detailed mechanism underlying LL-37 killing of *A*. *baumannii* is mostly unknown.

In this study, we examined the effects of LL-37 on *A*. *baumannii* and found that the effects were exerted in a LL-37 dose-dependent manner and related with the cell concentrations tested. When the cells (1 X 10^7^ cells/ml) in 750 μl of RPMI-1640 medium were used to examine the anti-bacterial effect, LL-37 above 5 μg/ml could effectively kill the cells (Fig 1). However, the cell adhesion (Fig 2A and 2B) and cell motility (Fig 2C and 2D) data are still valid beyond 5 μg/ml LL-37 concentration because a higher cell density (4 X 10^8^ cells/ml) and cell count (~1 X 10^9^ cells) were used in the adhesion and motility tests, respectively. Although LL-37-mediated inhibition of adhesion and biofilm formation in bacteria has been reported previously [41,42], this study is the first to report an inhibitory effect of LL-37 on bacterial motility. LL-37 is commonly secreted at mucosal surfaces at a concentration ranging between 2 to 5 mg/ml [43,44]. The effects of LL-37 on *A*. *baumannii* in our study were observed at the physiological concentrations in humans. Moreover, our study showed that BA-LL37 exhibited the most efficient killing of *A*. *baumannii* among the three tested AMPs (Fig 4A and 4B). The charges of LL-37, hBD3 and Hst5 in the physiological environment are +6, +11 and +12, respectively [45]. Both LL-37 and Hst5 have random coil conformations in hydrophilic environments and α-helical structures under hydrophobic conditions. In contrast, hBD3 has a β-sheet structure due to the presence of three intra-molecular disulfide bridges. How these structural differences among the three AMPs influence their ability to kill *A*. *baumannii* deserves further study.

Most studies suggest that AMPs act on Gram-negative bacteria through their surface LPS molecules [46–48]. However, several studies have emphasized the interaction between AMPs and OMPs. Outer membrane protein I (OprI) of *Pseudomonas aeruginosa* has been shown to be the target of cationic AMP [49]; this AMP can also interact with OmpF from *E*. *coli* [50]. In this study, far-western analysis revealed there were several LL-37-binding candidates; among them, OmpA was confirmed using an anti-OmpA antibody (Fig 5). This finding adds a pluripotent function to *A*. *baumannii* OmpA in addition to its roles in cytotoxicity, cell adhesion and immunomodulation. OmpA is an outer membrane porin protein and has an amino acid sequence that is highly conserved among Gram-negative bacteria [30]. In our study, BA-LL37 bound to the AbOmpA_74-84_ peptide (loop 2) but not the loop 4 peptide in a dose-dependent manner (Fig 6B). A hypothetical arrangement of the OmpA protein suggests that it repeatedly traverses the outer membrane in a cross-β structure, exposing the four loops to the outside [51]. Of these four loops, loop 2 appears to interact with the core carbohydrates of LPS. As shown in S3 Fig, an LPS defect was able to influence the bacteria-killing ability of LL-37, which implicates LPS as another target of LL-37. These results suggest the possible association and involvement of specific regions of OmpA and LPS as the mode of action of LL-37 against *A*. *baumannii*.

LL-37 not only exerts its antimicrobial effect by the formation of membrane pores leading to membrane disruption [6], but can also cross lipid membranes of host cells, resulting in gene/protein stimulation or a block of gene/protein expression [3]. In this study, the *ompA* deletion strain was more sensitive to LL-37 than the wild type strain (Fig 8A). Although the LL-37-binding target AbOmpA was absent, other OMPs might be bound by LL-37 (represented in Fig 5). Because OmpA exhibits low pore-forming function and permeability, with a pore size approximately 2 nm in diameter [52], LL-37 might bind to other OMPs with higher pore-forming abilities and permeabilities in the *ompA* deletion strain, resulting in increased cell death. Moreover, AbOmpA plays an important role in adhesion and biofilm formation in *A*. *baumannii* [23]. Fig 8B showed that the adhesion defect in the Δ*ompA* strain was not augmented as obviously as the defect in the wild type strain by the addition of LL-37. Therefore, we suggest that the LL-37-mediated adhesion defect may be explained by interference with AbOmpA.

LL-37 may bind to LPS with high affinity, but its bactericidal activity is not LPS-dependent. The increased sensitivity of LPS-deficient colistin-resistant *A*. *baumannii* to LL-37 has been demonstrated and ascribed to increased membrane permeability [17]. However, colistin-resistant isolates (due to mutations in the PmrB domains post-colistin treatment) induced cross-resistance to LL-37 [53]. Hence, we speculated that the LPS-defective strain with increased resistance to LL-37 (S3 Fig) might be a result of a PmrB mutation. While studying *P*. *aeruginosa*, Lin et al. proposed a model that suggested that the associated LPS and fatty acids of OprI were eliminated by AMP hRNase 7 treatment, followed by subsequent internalization of OprI with the invading hRNase 7. According to the results of our experiment, it is possible that LL-37 exerts its action on *A*. *baumannii* via OmpA binding in a manner that is similar to the results reported for *P*. *aeruginosa*.

There were some limitations to this study. First, the adhesion assay was performed on abiotic polystyrene plate whose characteristics were completely different from the cell surface *in vivo*. Second, the effects of LL-37 on *A*. *baumannii* were not demonstrated in human cell platforms. Finally, the contribution of LPS to LL-37 action in *A*. *baumannii* deserved further investigation. In conclusion, our study demonstrated that the human antimicrobial peptide LL-37 affected *A*. *baumannii* via binding to OmpA. We hope that this study can serve as a starting point to understand the complete mechanism underlying the effect of LL-37 on *A*. *baumannii*.

## Supporting Information

S1 Fig Anti-bacterial effect of LL-37 on clinical isolates of *A*. *baumannii*. The bacterial killing activity of LL-37 on two clinical isolates of *A*. *baumannii* was performed using spot assay, which also showed the anti-bacterial effect augmented with LL-37 concentrations increasing. The clinical strains are from our previous study [54].
(TIF)

S2 Fig Verification of the Δ*ompA* mutant construction. (A) RT-PCR was performed to detect *ompA* expression. Total RNAs were isolated from wild type and the Δ*ompA* mutant, cDNAs were synthesized and RT-PCR was performed. The absence of the *ompA* transcript (504 bp) was observed in the mutant strain.(B) OmpA protein expression was detected by Coomassie blue staining and western blot. OMPs of the wild-type and Δ*ompA* strains were extracted and subjected to SDS-PAGE. After transferring the proteins onto PVDF membrane, gel was stained by Coomassie blue and the membrane was blotted by anti-OmpA antibody. No OmpA protein expressed was detected in the Δ*ompA* mutant.
(TIF)

S3 Fig Sensitivity to LL-37 between the wild type and lipopolysaccharide (LPS) defect strain. (A) LPS of the wild type and the LPS-defective mutant was visualized by silver staining. LPS was isolated, subjected to a polyacryamide gel and analyzed by electrophoresis. Lane 1 was the LPS control from *E*. *coli*. (B) Sensitivity of the wild type and a LPS-defective strain to LL-37 was examined by spot assay. The wild type and LPS-defect strains were mixed with different concentrations of LL-37 for 1 hr, 10-fold serially diluted, and spotted on LB agar plate. The LPS-defect strain had better tolerance to LL-37 compared to the wild type.
(TIF)

S1 Table Bacterial strains, plasmids and primers used in this study. (PDF)
