# Supplementary figures and images for: OmpA Binding Mediates the Effect of Antimicrobial Peptide LL-37 on Acinetobacter baumannii
Source: PLoS One. 2015 Oct 20;10(10):e0141107. doi: 10.1371/journal.pone.0141107 (PMC4618850; doi:10.1371/journal.pone.0141107)

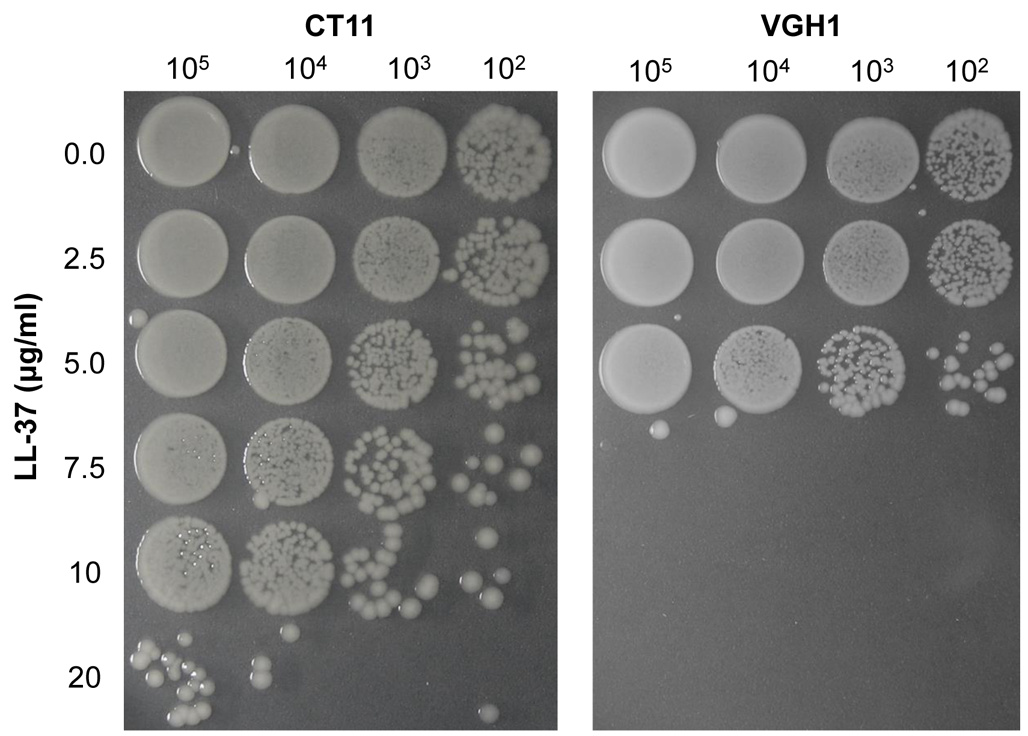

Supplement: S1 Fig — The bacterial killing activity of LL-37 on two clinical isolates of A. baumannii was performed using spot assay, which also showed the anti-bacterial effect augmented with LL-37 concentrations increasing. The clinical strains are from our previous study [54]. (TIF) [file pone.0141107.s001.tif]

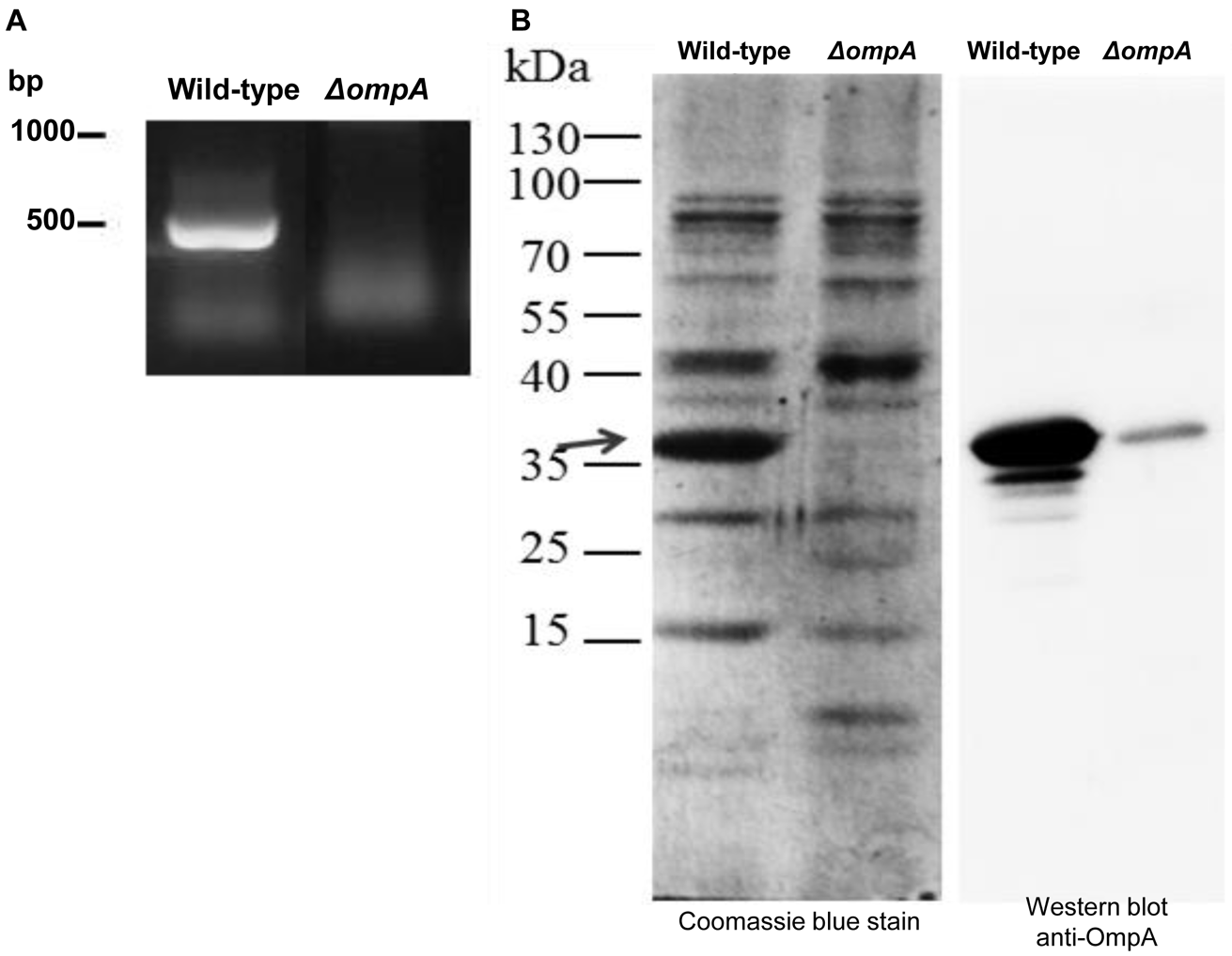

Supplement: S2 Fig — (A) RT-PCR was performed to detect ompA expression. Total RNAs were isolated from wild type and the ΔompA mutant, cDNAs were synthesized and RT-PCR was performed. The absence of the ompA transcript (504 bp) was observed in the mutant strain.(B) OmpA protein expression was detected by Coomassie blue staining and western blot. OMPs of the wild-type and ΔompA strains were extracted and subjected to SDS-PAGE. After transferring the proteins onto PVDF membrane, gel was stained by Coomassie blue and the membrane was blotted by anti-OmpA antibody. No OmpA protein expressed was detected in the ΔompA mutant. (TIF) [file pone.0141107.s002.tif]

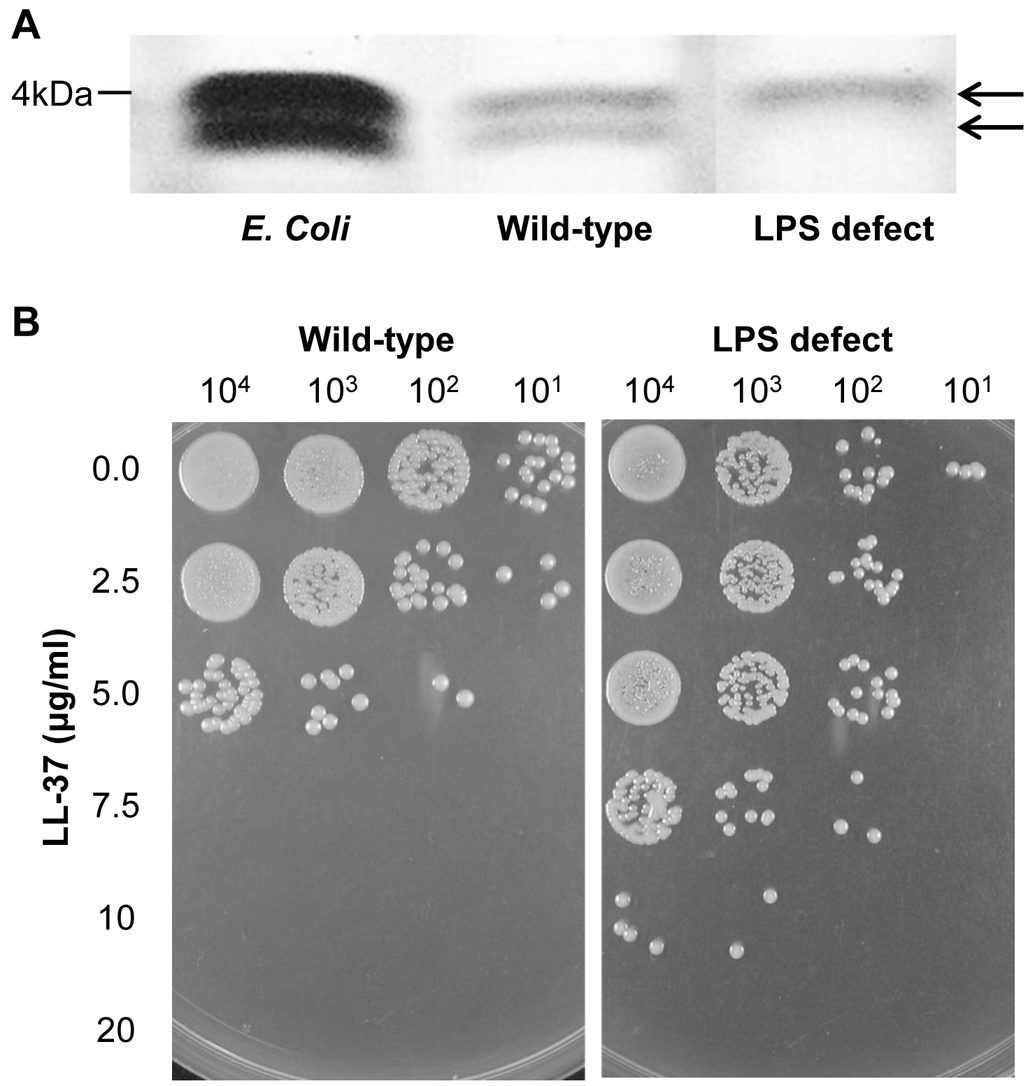

Supplement: S3 Fig — (A) LPS of the wild type and the LPS-defective mutant was visualized by silver staining. LPS was isolated, subjected to a polyacryamide gel and analyzed by electrophoresis. Lane 1 was the LPS control from E. coli. (B) Sensitivity of the wild type and a LPS-defective strain to LL-37 was examined by spot assay. The wild type and LPS-defect strains were mixed with different concentrations of LL-37 for 1 hr, 10-fold serially diluted, and spotted on LB agar plate. The LPS-defect strain had better tolerance to LL-37 compared to the wild type. (TIF) [file pone.0141107.s003.tif]
