# Supplementary material for: OmpA Binding Mediates the Effect of Antimicrobial Peptide LL-37 on Acinetobacter baumannii
Source: PLoS One. 2015 Oct 20;10(10):e0141107. doi: 10.1371/journal.pone.0141107 (PMC4618850; doi:10.1371/journal.pone.0141107)
Supplement: S1 Table — (PDF) [file pone.0141107.s004.pdf]

Bacterial strains, plasmids and primers used in this study

**Strains**		**Relevant feature(s)**	**Source or reference**
A. baumannii strains	ATCC 17978	Wild-type strain	ATCC
	ΔompA	Derived from ATCC 17978. *ompA* mutant obtained by kan^r gene replacement	This study
	17978CR (LPS defect)	Induced colistin resistant ATCC 17978	[1]
E. coli strains	BL21 (DE3) pLysS	F^- *ompT gal dcm* lon $hsdS_B(r_B^- m_B^-)$ λ(DE3) pLysS(Cam^r)	Novagen
	BL21 OmpA	BL21 (DE3) pLysS carrying plasmid pET23a-OmpA	This study
	XL1 blue	*recA1 endA1 gyrA96 thi-1 hsdR17 supE44 relA1 lac* [F' *proAB* $lacI^qZ\Delta M15$ Tn*10* (Tet^r)]	Stratagene
	S17-1 (ATCC 47055)	*thi pro hsdR hsdM recA*[*RP42-Tc::Mu-*Km*::Tn7* (Tp^rSm^r)Tra^+]	ATCC
	S17-1 ompA	S17-1 carrying plasmid pEX18Tc-OmpAUD-Kan	This study
**Plasmids**		**Relevant feature(s)**	**Source or reference**
pET23a		Subcloning vector with a T7 promoter, N-terminal T7 tag and C-terminal 6xHis tag; amp^r	Novagen
pET23a-OmpA		pET23a carrying *Acinetobacter baumannii* ATCC 17978 A1S_2840	This study
pEX18Tc		Suicide vector, containing *sacB*, Tc^r	
pEX18Tc-OmpAUD-Kan		pEX18Tc containing *ompA* upstream and downstream fragments joined by kan^r cassette	This study
**Primers**		**Relevant feature(s)**	**Source or reference**
ompA5'F		ATATGTCGACACCACCCATAGCAGTACGAG	This study
ompA5'R		ATATGGATCCTTGTTGTTCAAGCTCAGCCT	This study
ompA3'F		ATATGGTACCTACGATGTTGAGCACGAGGT	This study
ompA3'R		ATAAGAGCTCGACGCCATTGAACGGAA	This study

	AGT	
ompA int F	CCAACACAACAATGGCGGTA	This study
ompA int R	TCTACTACAGGAGCAGCAGG	This study
ompAExF	ATATGAATTCTACACTTTCCAAGACAGCCA	This study
ompAExR	ATATGCGGCCGCTTGAGCTGCTGCAGGAGCTG	This study
kanF	ATATGGATCCCCGGAATTGCCAGCTGGGGC	This study
kanR	ATATGGTACCTCAGAAGAACTCGTCAAGAA	This study

1. Kuo HY, Chang KC, Kuo JW, Yueh HW, Liou ML (2012) Imipenem: a potent inducer of multidrug resistance in *Acinetobacter baumannii*. Int J Antimicrob Agents 39: 33-38.
